# Defining avoidable healthcare-associated harm in prisons: A mixed-method development study

**DOI:** 10.1371/journal.pone.0282021

**Published:** 2023-03-15

**Authors:** Richard N. Keers, Verity Wainwright, Joy McFadzean, Kate Davies, Stephen M. Campbell, Caroline Stevenson, Thomas Purchase, Jennifer Shaw, Andrew Carson-Stevens

**Affiliations:** 1 Centre for Pharmacoepidemiology and Drug Safety, Division of Pharmacy and Optometry, University of Manchester, Manchester, United Kingdom; 2 NIHR Greater Manchester Patient Safety Translational Research Centre, Manchester Academic Health Science Network, Manchester, United Kingdom; 3 Suicide, Risk and Safety Research Unit, Greater Manchester Mental Health NHS Foundation Trust, Manchester, United Kingdom; 4 Centre for Mental Health and Safety, Division of Psychology and Mental Health, University of Manchester, Manchester, United Kingdom; 5 Division of Population Medicine, School of Medicine, Cardiff University, Cardiff, United Kingdom; 6 Epidemiology and Public Health Group, Division of Population Health, Health Services Research and Primary Care, University of Manchester, Manchester, United Kingdom; 7 Department of Public Health Pharmacy and Management, School of Pharmacy, Sefako Makgatho Health Sciences University, Pretoria, South Africa; 8 Independent Advisory Panel on Deaths in Custody, United Kingdom; Aalborg University and Aalborg University Hospital, DENMARK

## Abstract

**Background:**

Reducing avoidable healthcare-associated harm is a global health priority. Progress in evaluating the burden and aetiology of avoidable harm in prisons is limited compared with other healthcare sectors. To address this gap, this study aimed to develop a definition of avoidable harm to facilitate future epidemiological studies in prisons.

**Methods:**

Using a sequential mixed methods study design we first characterised and reached consensus on the types and avoidability of patient harm in prison healthcare involving analysis of 151 serious prison incidents reported to the Strategic Executive Information System (StEIS) followed by in-depth nominal group (NG) discussions with four former service users and four prison professionals. Findings of the NG discussions and StEIS analysis were then synthesised and discussed among the research team and study oversight groups to develop an operational definition of avoidable harm in prison healthcare which was subsequently tested and validated using prison patient safety incident report data derived from the National Reporting and Learning System (NRLS).

**Results:**

Analysis of StEIS incident reports and NG discussions identified important factors influencing avoidable harm which reflected the unique prison setting, including health care delivery issues and constraints associated with the secure environment which limited access to care. These findings informed the development of a new working two-tier definition of avoidable harm using appropriate and timely intervention, which included an additional assessment of harm avoidability taking into the account the prison regime and environment. The definition was compatible with the NRLS incident report narratives and illustrated how the prison environment may influence identification of avoidable harm and judgements of avoidability.

**Conclusions:**

We have developed a working definition of avoidable harm in prison health care that enables consideration of caveats associated with prison environments and systems. Our definition enables future studies of the safety of prison healthcare to standardise outcome measurement.

## Introduction

Improving patient safety in healthcare is a global priority, and there is evidence that patients are frequently exposed to avoidable, healthcare- associated harm [1–4]. Whilst progress has been made towards improvement by characterising avoidable patient safety incidents across primary and secondary care, the evidence base for prison settings remains limited [1, 4–6].

The Royal College of General Practitioners and Care Quality Commission have advocated for the concept of equivalence between primary care in the community and within secure environments including the prison estate. This means people in secure environments should have access to services and treatment that is consistent in range and quality as that available in the community [7] and to the rest of the population [8]. However, healthcare in prisons can be less than optimal/ inequitable [8–10]. Prison overcrowding and understaffing, and increasing rates of mortality, self-harm, and problems with substance misuse among this group have prompted renewed calls for improvement in healthcare quality in this setting [11–13].

Delivering safe and effective healthcare in the secure prison environment is challenging and requires consideration of factors unique to this context. For example, people in prison have poorer physical health, higher rates of mental illness and communicable diseases than the general population, with an ageing population that brings increasingly complex care needs [10, 11, 13–15]. For people detained in prison in England, the average age of death is 56 years [16]. Whilst people in prison may also access healthcare more often than those in the community [17, 18], health practitioners deliver care where strict security regimes may take precedent [19]. The structure of secure environments may restrict the delivery of health care within and outside prisons, for example through high rates of missed hospital appointments [20] which may be due to a lack of security escort. The influence of security also extends to medication management where patients may not be permitted to hold their own medications (i.e. ‘in-possession’ medication) and prescribing decisions may need to consider the risk of medication diversion or abuse [19, 21]. When considered alongside increasing numbers in custody and high prisoner turnover rates [22], it is important to focus on care quality and safety as a priority in this environment.

Core healthcare services commissioned by the National Health Service (NHS) in prisons are based on the primary care delivery model and their aim is to be equivalent to those cared for outside of prison [23]. Outside prison, people self-refer to access primary care through a dentist, pharmacy, a general practice doctor (GP) or nurse appointment at their registered practice. In prison settings, people are not registered with a general practice and their medical records remain with their home GP (if they had one). Prisoners do not have direct access to primary care services and instead have to request to see a doctor, nurse or pharmacist who may not have access to their pre-prison medical notes.

A critical step in the improvement of safety is adequate understanding and measurement of the problem(s) [24, 25]. Whilst studies conducted across healthcare settings have aimed to achieve this for numerous patient safety outcomes, differences in study definitions and methods have limited comparability and transferable learning possible from this collective knowledge. The World Health Organization has long championed core terminology to promote the generation of accurate, comparable data [26, 27]. To address this need, the research team has previously extensively characterised patient safety incident reports from primary care settings [28], developed a definition of avoidable harm in primary care (Box 1) and successfully applied this in a study involving retrospective review of 14,407 patient records [5, 29].

**Box 1. Definition of avoidable harm in the primary care**

“A patient safety incident could have been probably, or totally, avoided by the timely intervention of a healthcare professional (e.g. investigations, treatment, safety netting) and/or an administrative process (e.g. referrals, alerts in electronic health records, procedures for following up results) in accordance with accepted standards of evidence-based practice and/or clinical governance and/or the Bolam test*.”

*The Bolam test [1] refers to whether a healthcare professional can show that they acted in a reasonable and defendable way that a responsible body of healthcare professionals in the same field would regard as acceptable, taking into account evolving standards of care.

However, it remains unclear what types of healthcare-associated harm patients in prison experience that are unique to the setting, how these harms affect or change the existing definition in primary care [28, 29], what makes harm avoidable and to what extent harm occurs in prisons. Given the unique prison healthcare context and care equivalence challenges, this study therefore aimed to generate for the first time an operational definition of ‘avoidable harm’ within this environment to standardise outcomes for use in future behavioural, epidemiological and aetiological studies.

## Methods

This study was conducted as part of the ‘Avoidable harm in prison healthcare’ project (NIHR: PR-R20-0318-21001). This is a multi-phase project that aims to understand the frequency, burden and nature of avoidable harm in prison healthcare in order to improve patient safety.

### Design

**Fig 1:**
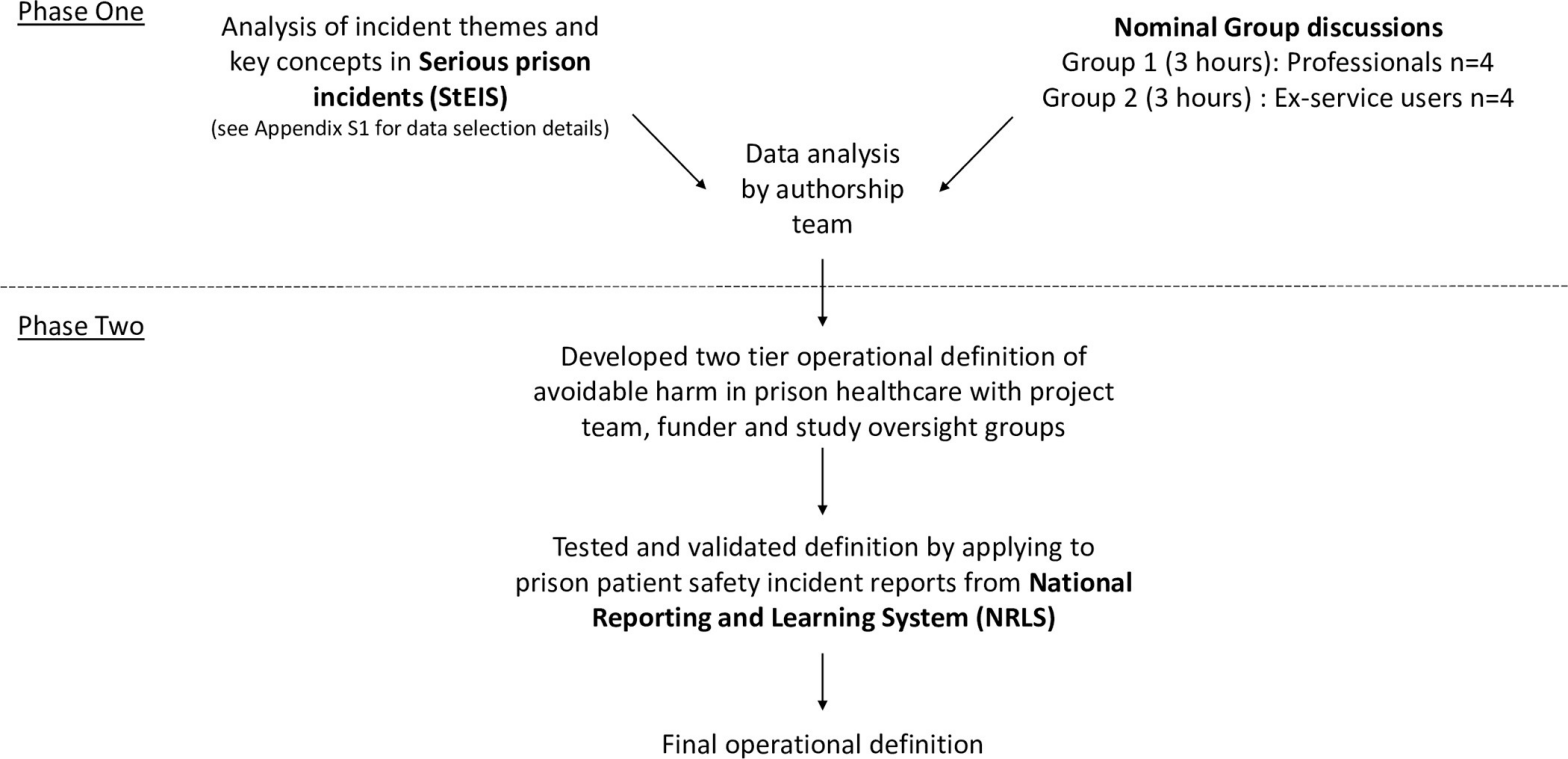
Summary of study methods.

A sequential mixed-methods approach comprising two main phases was used to develop and test the validity of an operational definition of avoidable harm in prison healthcare. This two phase process is summarised in Fig 1. The first phase incorporated synthesis of data arising from an analysis of serious incidents reported to the Strategic Executive Information System (StEIS) database and Nominal Group (NG) discussions with former service users and prison professionals designed to characterise and reach agreement on the types of avoidable healthcare-associated harm that could occur in prisons. The second phase of the study involved the wider ‘Avoidable harm in prison healthcare’ research team, study oversight groups and funder using the findings of phase one to generate an operational definition of avoidable harm in prison healthcare. This definition was then retrospectively applied to coded and thematically analysed prison patient safety incidents identified from the National Reporting and Learning System (NRLS) database for validation purposes.

### Phase one

#### Strategic Executive Information System (StEIS) incident analysis

Since 2002 serious incidents occurring in NHS organisations in England have been required to be reported and investigated [30]. These are defined as: unexpected or avoidable death of patients, staff, visitors, or members of the public; serious harm to patients, staff, visitors, or members of the public; a scenario that prevents or threatens to prevent a provider organisation’s ability to continue to deliver healthcare services; allegations of abuse; and/or, adverse media coverage or public concern about the organisation or the wider NHS.

Each report contains categorical information (such as incident type and location, and basic patient demographics) and a free-text commentary of what occurred, what immediate action was taken and a summary of the case. The reporting organisation is later required to complete further free-text fields detailing the investigation carried out, root causes identified, and lessons learned following the incident. A description of the database and the content of reports has been documented previously [30].

Fig 1 in S1 Appendix summarises the process of selecting StEIS reports filed between 1 April 2002 and 31 March 2013 that originated from prisons (n = 2046). Due to resource constraints, an experienced mixed-methodology researcher with expertise in Human Factors and incident reporting (ACS) manually reviewed a convenience sample of 151/2046 (7.4%) of the most recent reports with sufficient free text data concerning incident origins and improvement recommendations. The narrative of these 151 reports was then deconstructed through inductive open coding to represent their meaning [31] before themes based on this coding were generated and collated into a framework. Anonymised case vignettes were then created for review by the wider ‘Avoidable harm in prison healthcare’ research team which illustrated the thematic framework. These vignettes were used alongside findings from the next part of phase one (see below) to guide the development of the tailored avoidable harm definition in prison healthcare described in phase two of the project.

#### Nominal Group (NG) discussions

The Nominal Group (NG) technique was used to explore consensus on what kinds of avoidable harm related to prison healthcare occur, why and under what circumstances. This is a mixed methods consensus-building approach which has been described in detail elsewhere [32, 33]. Consensus building methods such as NG discussions have been previously used to generate consensus on a wide range of patient safety topics, including avoidable harm definitions, care transitions, and prescribing safety indicators [29, 34–36]. Organisation and recruitment for the NG discussions was led by VW and CS. Participants were recruited for two NG discussions: one service user group of former prisoners and the other with prison professionals (including clinical [e.g., doctors, psychiatrists, nurses, pharmacists] and non‐clinical roles [e.g. commissioners]) with at least three years’ experience in secure environments and who had knowledge/experience of patient safety in prison (i.e., experience in medicine management/safety and/or experience of prescribing safety and quality). Recruitment took place through the professional networks of the research team. All participants provided written, informed consent immediately prior to the discussions beginning and travel reimbursement was offered to all participants. Service user participants were provided with a £75 shopping voucher for taking part.

Data collection took place during December 2019 in private meeting rooms at The University of Manchester, and each NG discussion lasted three hours. In total, eight participants took part across the two groups: four former service user participants (two males and two females) and four professionals (one male and three females). All participants in the service user group were also members of a service user stakeholder oversight group for the wider ‘Avoidable harm in prison healthcare’ project. Professional participants comprised a prison healthcare manager, prison pharmacist and nursing and commissioner representatives for health and justice services in England. Four authors (RNK, SC, CS, VW) were present during one/both of the NG discussions, with RNK leading the sessions and CS taking field notes. Following a general introduction and setting of ground rules in accordance with established NG methodology [33, 37–40], participants were then asked to consider the nominal question: ‘*What patient harms are most likely to occur in a prison healthcare setting and how avoidable and severe are they*?’. Participants then individually generated thoughts and ideas in response to the nominal question, before calling out their ideas for the facilitators to display to the group in ‘round robin’ fashion. A discussion of these ideas was then facilitated for approximately one hour, where ideas were clarified, refined, and grouped into themes by the facilitators. Finally, participants were asked to select and rank their ‘top five’ resulting ideas, presented in order of importance.

Anonymised transcripts of NG discussion recordings were triangulated alongside typed researcher field notes and data generated by participants (ranking and sticky notes) for thematic analysis [41]. Authors VW and CS led on the analysis of the NG data, with RNK and SC independently reviewing the thematic framework. All authors met to discuss the analysis and reach agreement on the overarching themes, examples of harm and where consensus was reached in the ranking of ideas. The findings of the NG discussions were then used alongside the thematic framework arising from the StEIS analysis by the wider ‘Avoidable harm in prison healthcare’ research team to develop the avoidable harm definition in phase two, as described below.

### Phase two

#### Developing and reaching consensus on the avoidable harm definition

Thematic frameworks generated from the StEIS and NG analyses in phase one were reviewed by the authors in order to establish what prison healthcare related harms are (including any overlap with overall prisoner wellbeing), characterise the types of harms and explore what makes them avoidable. The author team includes a diverse group of health professionals (general practitioners [GPs]), pharmacist, forensic psychiatrist) and academics with backgrounds in patient safety and psychology. The nature of these avoidable harms were then assessed to determine the extent of alignment to our established primary care definition of avoidable harm [5, 29], with a focus on what made validity and/or application of this definition challenging in the prison context.

Findings arising from this assessment by the authors were then presented and discussed in a stepwise process as follows with wider teams in order to develop and agree the final avoidable harm definition for prison healthcare. At all times the discussions focused on how the unique prison context was different to primary care, and how this influenced judgements on harm avoidability in relation to the established primary care definition.

1. The wider ‘Avoidable harm in prison healthcare’ project team, a multi-disciplinary group comprising academics specialising in patient safety, forensic psychiatrists, GPs, sociologists, substance misuse specialists, psychologists, pharmacists, and mental health professionals. Patient and public representation was also present in the group;
2. Study oversight stakeholder groups. This includes a service user group comprised of four former prisoners recruited via existing networks, social media and third sector organisations alongside a study advisory group of 23 individuals comprised of prison GPs, prison policy makers/commissioners, senior prison leaders/managers, prison quality leads and academics with backgrounds in health economics, patient safety and criminology. These groups convene independently at quarterly intervals throughout the project to monitor and evaluate the progress of the wider study and to both hold the study team to account and help problem solve as issues arise.
3. The research project funder (National Institute for Health and Care Research (NIHR))/Department of Health and Social Care. As the funder and policy customer who commissioned the wider study, the research team liaise with representatives from these organisations to provide regular updates on the study and to monitor and evaluate progress according to key milestones. In addition, representatives for both the funder and DHSC attend the stakeholder advisory group.

#### Definition validation: National Reporting and Learning System (NRLS) incident report analysis

The agreed operational definition of avoidable harm in prison healthcare was then applied to anonymised patient safety incident reports identified from the National Reporting and Learning System (NRLS), now called the Learn from Patient Safety Events (LFPSE) service. The NRLS database was established in 2003 and has been integral in supporting improvements within patient safety. NRLS data has been, and will continue to be, extensively utilised for investigating patient harm across the healthcare continuum [42–46]. Prison-related incident reports were therefore deemed an appropriate dataset with which to validate the definition of avoidable harm in prisons. A patient safety incident is defined as “any unintended or unexpected incident which could have, or did, lead to harm for one or more patients receiving healthcare” [47]. NRLS incident reports contain structured information that includes location, free text describing the incident, contributory factors, and actions to prevent reoccurrence. As such it allows the categorisation of information contained in these reports and exploration of common patterns.

An analysis of incidents occurring in English prisons and remand centres reported to the NRLS between April 2018 and March 2019 which contained contributory factors (n = 1529/4112 in total, 37%) were used as the dataset for applying the avoidable harm definition. These incidents were first coded by two clinical academics (JM and KD), and codes were assigned from the classification system outlined by the Primary Care Patient Safety (PISA) Research Group which has been utilised previously [48–50]. Codes were applied systematically and chronologically adhering to rules of the Recursive Model of Incident Analysis [51] to capture incident type, outcome and contributory factors. The codes were assigned to reflect explicit statements by incident reporters, and no inferences were made by the coders. A thematic analysis followed, using an *in vivo* approach in order to determine themes and patterns to understand the context, event sequence and incident antecedents.

The avoidable harm definition generated by the research term was then applied to the thematic data and incident narratives to consider (a) whether it could be used to clearly identify instances of avoidable harm, and (b) whether the influence of the prison regime and/or environment informed judgements concerning the avoidability of harm. Examples of incident report classification using the new avoidable harm definition were discussed amongst the research team (RNK, ACS, JM, KD, SC, VW) and an iterative process followed to refine classification criteria until consensus was reached.

### Study approvals

For the StEIS and NRLS data analysis, a data sharing agreement was created between Cardiff University and NHS Improvement/England (DSA 5131) and ethical approval was provided by the Cardiff University School of Medicine Ethics Committee (SMREC 20/83). For the NG discussions, ethical approval was provided by the University of Manchester Research Ethics Committee (2019-7330-11582).

## Results

### Summary of Phase one: StEIS incident reports and NG discussions

The NG discussion and StEIS analysis identified a range of avoidable harms in prison health care, including examples relating to both physical and psychological harm, for example arising from delayed medical appointments or medicines issues related to bullying or diversion. The StEIS analysis key themes with supporting vignettes and illustrative participant quotes, alongside supporting key themes from the NG discussions are presented Table 1 and Box 1 in S1 Appendix. Areas of agreement concerning factors underlying the avoidability of harm events across the StEIS and NG thematic analyses included health care delivery issues and prison environmental constraints.

Prison health care delivery issues were reported in StEIS data (n = 80) as failure to follow evidence-based practices (n = 27), deficient initial assessments when entering prisons (n = 23) and when risks or changes in patient behaviour prior to suicide were not detected (n = 15). NG discussions also identified how complex/vulnerable patient factors may have impacted on how they were perceived and given care by prison staff; for example how behavioural/medical issues that were perceived as negative by staff drove responses to how it was dealt with at the time. NG thematic analyses also revealed cultural issues concerning a perceived lack of standard of equivalence with community services with health care needs being seen as secondary to security.

Prison environmental constraints were a reported antecedent to avoidable harm events across the StEIS (n = 13) and NG data sets, including issues relating to staffing levels, experience and continuity alongside rigid prison rules which contributed to limited access to treatment for example through delayed appointments or access to medication. Other themes reported included medication incidents arising from StEIS data (n = 45) which were reported to commonly arise due to the timely access to prescribed medications (n = 29) and issues with methadone prescribing or administration (n = 13).

### Summary of Phase Two: Reaching consensus on avoidable harm definition and validity testing

#### Developing and reaching consensus on the avoidable harm definition

Following initial discussions of the phase one study findings by the authors, the types of avoidable harm in prison health care were considered to be adequately identified and described using the existing definition of avoidable harm for community-based primary care settings developed previously by the authors (as shown in Box 1) [29]. It was agreed that potential cases of avoidable harm should be judged against this definition with users considering whether they are satisfied to state that “*the staff/prison could have done no more*” in reaching their decision on whether the harm was, on the balance of probability, avoidable.

However, the authors identified from the review of phase one findings that this primary care definition did not alone adequately account for the prison structures and contextual environment (e.g. security regime) and how this could influence the ability of staff to intervene and therefore influence judgements on harm avoidability. Therefore, following further discussion and contextualisation of these findings by the wider ‘Avoidable harm in prison healthcare’ study team, study oversight groups and the study funder, it was agreed that judgements on the avoidability of patient harm should therefore be made on a two-tier basis:

- **Tier 1:** assessment of whether more could have been done, with equivalence to how community cases would be assessed (see Box 1). Therefore, a judgement is made regarding avoidability, without consideration of the caveats introduced by the prison regime, system, and environment.
- **Tier 2:** With prison-experienced General Practitioner (GP) input, a further assessment should be made about whether or not the prison could have done more, considering the restrictions imposed by the regime and environment (i.e., to aid an understanding of what aspects of the prison environment may have contributed to harm cases [e.g., resource and service availability, lack of prisoner autonomy to coordinate appointments etc.]).

In rating the avoidability of harm, it was decided that a six-point system developed to assess the preventability of deaths in English acute hospitals (and previously used in a study of avoidable harm in the community [5], would be adapted for use [52]. The results of the NG discussions and the analyses of NRLS data supported this rating of avoidability of harm.

1. Totally unavoidable (Virtually no evidence of avoidability)
2. Unavoidable (Slight to modest evidence of avoidability)
3. Possibly avoidable (Less than 50–50, but close call)
4. Probably avoidable (More than 50–50, but close call)
5. Probably avoidable (Strong evidence of avoidability)
6. Totally avoidable (Virtually certain evidence of avoidability)

#### Guiding principles for the avoidable harm in prison healthcare definition

Differences compared to the primary care setting definition (Box 1) related to how the provision of healthcare services interfaced with the structures and processes in a prison setting and restrictions imposed by the environment; professionals reported a tension between managing security risks and healthcare need, for example a prisoner considered very high-risk needing to leave the prison for a non-urgent medical procedure can present a difficult challenge for the prison, needing not just to consider healthcare need, but the wider security risks. The definition recognises that even when structures and processes are in place to intervene, unique prison contexts such as security can stymie their access or use which may precipitate healthcare related harm (e.g., daily care transfer thresholds to hospital appointments, opportunity to collect daily dispensed medications or an overall imperative to prioritise security).

In the context of this working definition, harm was agreed across the StEIS and NRLS analysis along with the NG discussions to constitute either physical or psychological nature with the latter including emotional distress as a result of a healthcare issue. For instance, former service users spoke about how inappropriate prescribing could lead to issues of violence and bullying, giving examples of prisoners swapping medications between themselves or having their medication taken from them by other prisoners. The research team also concluded that *harm* must never be classified as unavoidable due to the prison setting alone and should be judged on the basis of Tier 1 presented above. The additional information gathered as part of Tier 2 is designed to help users of the definition to understand prison specific constraints/explanations for the harm that may help inform decision-making processes of avoidability. This definition and guidance builds on the definition of avoidable harm in primary care in Box 1 [29], taking into account the unique prison environment and in particular, the challenging interplay between the prison regime and security and healthcare provision in relation to decisions concerning whether the harm was avoidable.

#### Validity testing of avoidable harm definition

Specifically, 1529 patient safety incidents from the NRLS dataset were retrospectively reviewed and the avoidable harm definition conceptually applied. Vignettes were created and discussed as a team to show the clear delineation between both tiers, drawing on prison experiences and knowledge to consider Tier 2 differences. The working definition was found to be compatible with the incident report narratives, both in identifying cases of avoidable harm and in capturing the context of the prison environment in facilitating judgements concerning incident avoidability according to the Tier 1 and Tier 2 model. To illustrate this, Tables 2–4 in S1 Appendix contain examples of how judgements concerning the avoidability of harm may differ depending upon whether a patient safety incident (relating to cardiovascular disease, mental health illness or medication) is considered under Tier 1 or Tier 2 of the avoidable harm definition, whilst also reflecting the level of detail that may be required in medical records to make such judgements.

## Discussion

### Main findings

The operational definition for avoidable harm in primary care developed for community settings (Box 1) [29] was deemed relevant to prison settings but with important contexts and caveats. As Donabedian stated in 1966 [53], good structures and processes of healthcare provide enhanced opportunities for, but do not guarantee, good outcomes. The availability of structures (staff time and expertise, medication, equipment etc.) and processes (delivery of care) does not guarantee the opportunity to apply them to optimum benefit, as they can be affected by the prison context. These include prison environment constraints, documentation availability, prison rules, unstable or changing workforce, inability to conduct an initial assessment on entering prison due to staff unavailability or time of day and so on (Table 1 in S1 Appendix). Consequently, the definition of avoidable harm we have developed and validated requires judgements about avoidability of harm within the prison estate, which must be informed by an understanding of the relationship between healthcare services and providers in prisons and the wider team and structures responsible for overall safety, wellbeing, and security.

Contexts and barriers unique to, or aggravated by, a prison setting compared to community settings identified in this study included issues at a micro, meso and macro/systemic level (see Tables 1–4 and Box 1 in S1 Appendix). Examples of micro level issues included a lack of or poor communication, lack of control or autonomy over self-care, failure to follow up investigations or referrals and inadequate monitoring of individual patient physical and mental health. Meso or organisational contexts referred often to either cultural or systemic ways in which a prison is run, such as medication management at set times only, delays in care due to lack of monitoring procedures, inappropriate management or failure to follow guidelines due to the absence of an agreed protocol, staff shortages, turnover of staff or lack of appropriately trained staff, staff behaviour and attitudes due to prison culture, training issues and an expectation for prisoners to self-manage care. At a macro level, diverse and multiple providers mean a lack of accountability and continuity and there is often a focus on security as an imperative over healthcare.

The recommendations raised to contextualise the definition within the prison environment resonated with the advocacy of the concept of equivalence between primary care in the community and within secure environments including prison estate due to prison healthcare previously being reported as inequitable [9, 54]. In this respect, equivalence relates to access to services and care that are “at least consistent in range and quality (availability, accessibility and acceptability) with that available to the wider community in order to achieve equitable health outcomes” [7]. The analyses of StEIS, NRLS data and NG discussions highlighted specific prison environmental and cultural constraints and contexts that mean those in prison can experience prison estate specific but avoidable health care related harm. These can be due to failures of, or delays in, continuity and coordination of care as well as opportunities to access care or be assessed and/or monitored. As in community settings [2], medication related incidents were most commonly reported in the StEIS and NRLS analyses. The NG discussions often showed the resultant impact on both the physical and also emotional health of patients, with the most frequently reported outcome within NRLS data involving self-harm and mental health deterioration.

The reasons for avoidable harm in prisons are many and complex and our results provide signals that these may be due to structural or cultural norms or finances or workforce availability [5] that can create difficulty embedding or applying agreed healthcare protocols or behavioural norms. While this can be directly the result of the priority of security or prison rules, contingency changes to healthcare delivery to adapt and deliver equivalence can be lacking. This may be particularly relevant for contingency strategies to address delays caused by security issues, inappropriate management due to staff without appropriate training, lack of opportunities to identify risk, or inadequate initial, ongoing assessment or follow-up.

Other issues, while aggravated by the prison context and factors such as poorly trained or changing staff, are avoidable and do undermine equivalence. For example, the probability of harm can be compounded by a failure to follow local protocols or national guidelines (Table 1 in S1 Appendix). In addition, in the NG discussions both staff and ex-prisoners referred to cases where patients reported poor quality of care because they were not treated with dignity or were not listened to. Interpersonal care underpins the professional-patient relationship, with communication and knowledge of the patient the cornerstones of building trust in healthcare and especially primary care [55] and hence a fundamental priority for an equivalence with community settings, which can be lacking as shown in (Box 1 in S1 Appendix). In identifying potential key underlying antecedents to avoidable healthcare-related harm our findings therefore present a foundation from which future research can explore this aetiology in greater depth, which in turn can support the development of remedial interventions.

Other factors contributing to avoidable harm are modifiable and are not unique to healthcare but the way prisons are run. Prisons individually have limited budgets and staffing. This means that even where the culture privileges healthcare where possible and staff are trained and provide care within the limitations of the setting, systemic issues can stymie healthcare. These include poor information technology, security rules for ambulance release or medication management, staffing levels, the availability of appropriately trained staff and prison overcrowding [15]. For example with prison electronic health records it has been reported how limitations affecting interfacing with external systems may delay verification of medication records if the information needed is not available when required [19], and there are ongoing discussions across UK healthcare considering how inter-operability and sharing of health information (including with patients) could address challenges such as these [56–58]. However, such issues can have deleterious consequences on physical and psychological morbidity, high rates of mortality, self-harm, and substance misuse [10]. If one considers that quality of care can be seen as a focus on individual care in parallel with and in the context of population care [59], many healthcare related harm incidents may derive from a lack of individual care.

Our findings reinforce the need for healthcare improvement in this setting, both generally and specifically given the changing demographics of the prison population with increasing numbers of aging or elderly patients [9, 11, 54, 60]. Considering avoidability of patient safety incidents is vital alongside other characteristics such as their severity for considering priorities and directing improvement efforts [4]. We envisage our avoidable harm definition can be instrumental in addressing this challenge by facilitating consistency and rigour in the conduct of relevant epidemiological and aetiological studies as observed for primary care settings [5, 29]–the research team aim to apply this definition to an epidemiological study currently underway using case note review to explore the burden of avoidable harm in prison healthcare. Such rigour and consistency of methodology is required to facilitate future comparable studies of the safety of prison healthcare, but also to estimate the frequency and genesis of disease and illness within prisons to standardise outcome measurement in order to investigate and address the nature and causes of avoidable harm more systematically.

The impact on prison staff as second victims of patient safety incidents [60, 61], is also an important issue that can mean experiencing significant personal and professional distress. This can result from being involved in an error, witness to prisoner emotional distress or self-harm or not being able to provide care clinically indicated due to ambulance/accompanied visit barriers etc. This has potential implications for staff turnover, and may result in a more transient and less-experienced workforce which reduces continuity of care and the experience needed to identify and address possible avoidable harm.

This study has a number of strengths. The definition of avoidable healthcare-associated harm is grounded in stakeholder experience and evidence of patient safety incidents in prison practice and has been contextualised by researchers experienced in creating and applying avoidable harm definitions in primary care contexts. Furthermore, it was developed using a sequential process where data iteratively informed subsequent stages to support validation of data generated between them. We also employed retrospective application of our definition to a variety of safety incidents describing contributory factors to confirm its validity. Nevertheless, some limitations are worthy of mention. Whilst StEIS analysis enabled the team to initially appreciate high-level differences between the community and prison context, a convenience sample of approximately 50% of the most recent available reports were analysed and more detailed examination of safety reports is needed. In addition, although clear areas of agreement existed between the two NGs and they included a range of stakeholders, the groups are not representative of all professional and patient perspectives. The groups also focused on describing the underlying antecedents of harm in prisons rather than its nature. However, this directly informed the constructs underpinning the two-tier system by highlighting the key differences between prison health care and other settings in what makes harm avoidable.

The NRLS data contained a large volume of incident reports, spanning across a breadth of incident types. Whilst we can acknowledge that there is potential for reporter and selection bias, to our knowledge there is no other routinely available data source to carry out such a task. Further case note review will now allow us the opportunity to further appraise its utility through a different lens on the safety phenomenon.

## Conclusion

Patient safety deficits and harms occurring in the community can occur also in prison settings. We have modified and validated a definition of avoidable healthcare-related harm in primary care for prison settings that reflects additional avoidable healthcare related harm that arises or is compounded by prison specific environmental and cultural constraints, barriers and contexts. Some of these harms relate to modifiable and avoidable poor quality of care pertinent equally to primary care or prison settings, such as not following guidelines and not listening. However, others may present and be managed differently and are compounded by prison specific barriers such as staffing, financing and security precedence. Our definition is designed to account for the contribution of the secure environment to avoidable harm judgements. There is a need for high-quality epidemiological and behavioural studies to establish a baseline and comparative data, identify issues amenable to improvement and develop setting specific interventions to reduce avoidable harm to patients. Because there are “aspects of care provision within secure settings that require a different approach or service model than would otherwise be available in the wider community… ‘equivalence’ does not mean that care provision in secure environments should be ‘the same’” [7] but that “quality of care is at least equivalent to the rest of the population” [8]. This requires quality and avoidable harm to be defined and measured in standardised ways in prison settings so they can be compared between secure settings and then to that in the wider community. This paper provides the basis for a definition of avoidable harm for future studies in the prison context, and will now be tested in a large, comprehensive, case note review study in eighteen prisons in England.

## Supporting information

S1 Appendix Supporting files. (DOCX)
