# Supplementary material for: Defining avoidable healthcare-associated harm in prisons: A mixed-method development study
Source: PLoS One. 2023 Mar 15;18(3):e0282021. doi: 10.1371/journal.pone.0282021 (PMC10016636; doi:10.1371/journal.pone.0282021)
Supplement: S1 Appendix — (DOCX) [file pone.0282021.s001.docx]

**Defining avoidable healthcare-associated harm in prisons: a mixed-method development study**

Short title: Defining avoidable harm in prison healthcare

Richard N Keers, Verity Wainwright, Joy McFadzean, Kate Davies, Stephen M Campbell, Caroline Stevenson, Thomas Purchase, Jennifer Shaw, Andrew Carson-Stevens

**S1 Appendix: Supporting files**

**Figure 1: Sample selection flow diagram for phase one analysis of StEIS data**

**
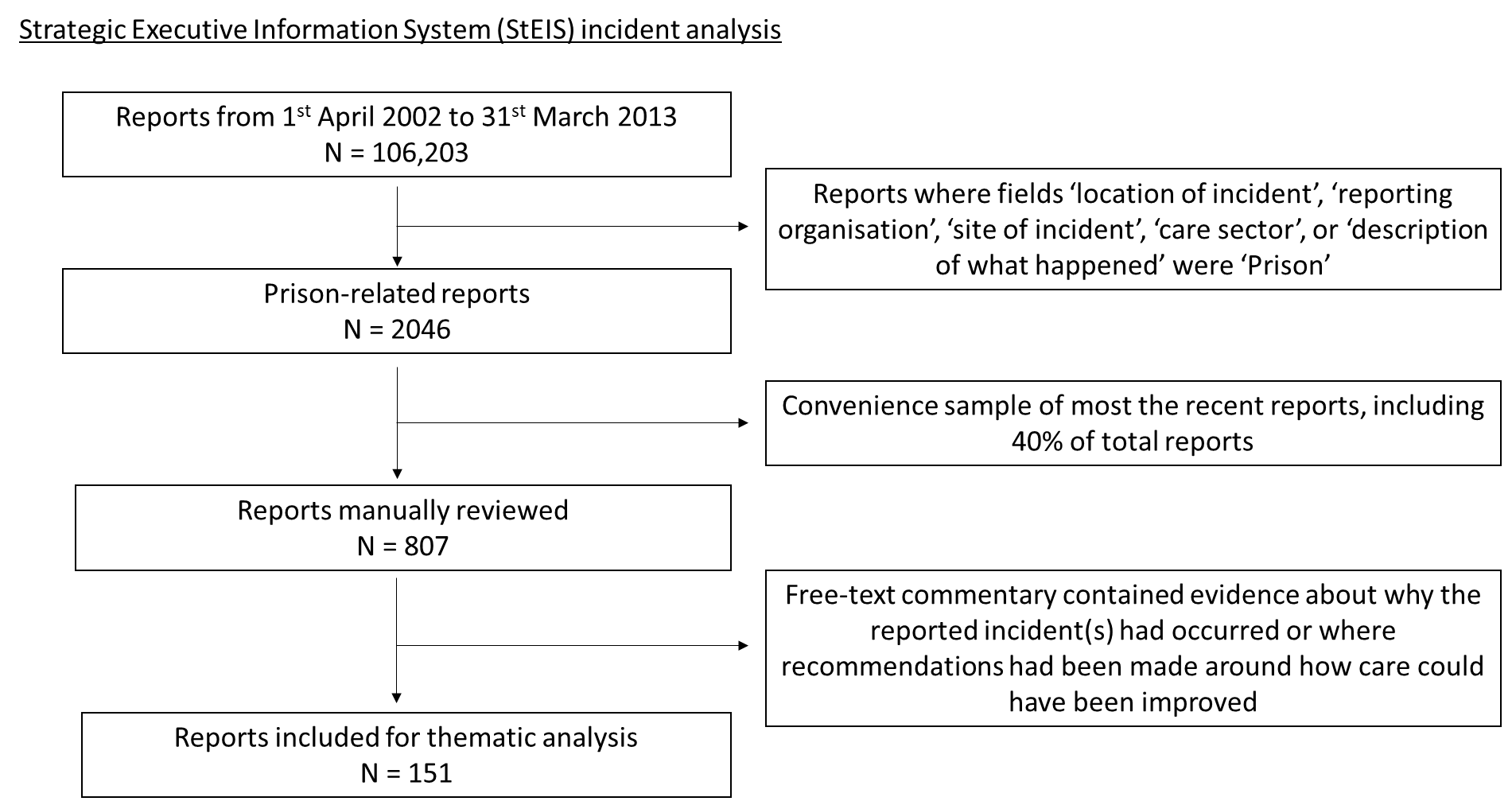
**

**Table 1: Summary of StEIS incident categories**

| **Category,** theme – *definition* | **Frequency, n (%)** | **Vignettes** |
| --- | --- | --- |
| **Clinical management of patients** | **80 (53%)** | *Vignette 1. Patient taken an opiate overdose. Paramedic called to prison and gives patient naloxone and then left the patient under the care of nurses in the prison. The patient subsequently deteriorated, and the same paramedic crew transported the patient to hospital. Following a period in ICU, the patient died.*  *Vignette 2. Patient with chest pain at night. Reviewed by nurse and advised to see doctor in the morning. GP arranged emergency admission at the arranged appointment, but the patient collapsed whilst waiting for an ambulance and had a cardiac arrest and died.*  *Vignette 3. Patient with chest pain in the middle of the night. Reviewed by nurse and administered Gaviscon. For review by the GP in the morning. Unresponsive in the morning and in cardiac arrest. Failed CPR and died in prison.* |
| Inappropriate management – *not following evidence-based practices* | 27 |  |
| Initial assessment when entering prison from another setting – *deficient or absence of essential health checks carried out* | 23 |  |
| Identification of suicide risk – *inability to identify risks / change in behaviour prior to suicide* | 15 |  |
| Delayed management and diagnosis – *lack of timely healthcare delivered to patient* | 13 |  |
| Failure to follow up investigations or referrals –*delayed or incomplete review of important tests or referral for specialist care input* | 2 |  |
| **Medication-related incidents** | **45 (30%)** | *Vignette 4. Patient with epilepsy arrived at prison. Noted to take three types of anti-epileptic medication. He transferred wings and drugs got left behind. Did not receive medications on day of arrival, and drugs dispensed the following day. Had a seizure and required A&E visit due to injuries.*  *Vignette 5. Unwell patient admitted to hospital. Prison faxed wrong records and non-diabetic patient administered insulin for 48 hours. Two major hypoglycaemic event ensued. Diabetic specialist nurse attended patient inquiring about his diabetes to which the patient replied “I am not diabetic”.* |
| Medication access – *timely access to medications intended by the prescriber* | 29 |  |
| Methadone-related – incidents arising relating to the prescription and administering of opiate replacement | 13 |  |
| False identify – patient claiming the identify of another prisoner to obtain medications prescribed to another inmate | 3 |  |
| **Prison environment constraints** | **13 (9%)** | *Vignette 6. Patient with chest pain required a blue light ambulance to hospital. Reception staff led to believe it was a normal (non-emergency) transfer to A&E and held up 45 minutes whilst the staff completed paperwork. The patient was handcuffed throughout and was searched. Patient deteriorated whilst waiting for reception staff to give security clearance for transfer to A&E.*  *Vignette 7. Patient stabbed and requiring immediate transfer to hospital. Paramedics attended scene and stabilised patient. Patient did not leave prison as quickly as needed due to security issues.*  *Vignette 8. A patient made a significant self-harm attempt. Despite bleeding profusely, the patient refused to go to A&E. Staff were unclear around how to manage the refusal of emergency treatment.* |
| Staff-related issues *– the behaviours of prison staff including violence towards prisoners impacting on healthcare received* | 6 |  |
| Prison rules – *limitations on timely access to healthcare services externally and for permitting external providers into prisons (e.g., ambulances)* | 4 |  |
| Restraint – *the physical restraint of a prisoner contributing to an incident* | 2 |  |
| Information technology *– inability for timely access to records* | 1 |  |
| **Suicide on release from prison** | **7 (5%)** | *Vignette 9. Patient previously open to mental health team and had several triage assessments in the last year, since their discharge from prison, has been found deceased at home. Had been reported as a missing person, found by police hanging from ligature -suicide note present.* |
| **Documentation availability and practices** | **6 (4%)** | *Vignette 10. Patient had self-harmed whilst in custody and doctor noted low mood. Assessment, Care in Custody and Teamwork (ACCT) document opened and doctor advised close observation. Three weeks later staff downgraded the level of observation. Seen by GP two weeks later for presumed heartburn; no review of mood undertaken. Two weeks following the GP review, the patient had hung himself in his cell.*  *Vignette 11. Patient assessed by mental health team following an attempted suicide. One month later, he returned to the general prison wing and staff felt he was positive with no concerns. At breakfast, he refused to leave his cell, and was found dead several hours later.* |

**Box 1: A summary of illustrative quotes supporting key themes from the NG Discussions informing harm avoidability**

***Theme 1: Complex/vulnerable patient factors***

*“.. if violence is started or instigated by someone not being listened to, and they don't have the skills to articulate, you're not believing me and I'm in need, and they act violently, these things have repercussions for release, for intervention programmes, for family ties, for visits, for phone calls, for money.”* (Service user participant)

*“What’s springing to my mind is somebody who’s possibly on the autistic spectrum but people see it as a behaviour to get what they want, a challenging behaviour that’s being done on purpose or for their own gain, and yet actually it’s actually somebody who’s not coping in the prison environment and doesn’t know how to cope or communicate that, so they’re seen to be more of a challenging…”* (Professional participant)

*“.. nobody ever did a mental health assessment on this person because they were so violent that [they] were continually told you can’t go in, it’s too risky… so there was no mental health assessment, and the individual then completed their suicide.”* (Professional participant)

***Theme 2:*** ***A lack of standard of equivalence with community services***

*“.. when I was going in prison and … told the doctor the medication that I was on, …he turned around and said, no, I'm sorry, you can't have that medication, he didn't offer me anything else… I said if I don't have that medication by Sunday… I'll be ill… He said you have to wait to see the psychiatrist who will be here Monday or Tuesday. By Saturday night, I'm climbing the walls..”* (Service user participant)

*“.. the system means that you'll get your appointment slip the day after your appointment, or that your application gets lost in the process, you could be waiting a long time… miss a really important appointment..”* (Service user participant)

*“..I think there’s a lot of tension between even the most caring of prison officers and governors and actually healthcare.. the very worthy and commendable expectation amongst healthcare practitioners that everyone has the right to care no matter what their offending behaviour is..”* (Professional participant)

***Theme 3: The capacity and culture within a prison***

*“The issue with prison regime, they have a trump card… [it’s] security each time. And you can't argue with that… the utilitarian principle of security… we need to keep everyone secure… How can you argue with that? So the regime is sacrosanct, you know. And then… you go and talk to healthcare staff and they go, yeah, we're trying… but… we come up against the regime.”* (Service user participant)

*“I think it's important to say that the bad practice of healthcare is mostly a consequence of the prison system and its restrictions. But there are some issues, of course, with staff competencies and compassion, and manner with patients and their judgements.”* (Service user participant)

*“.. we’ve got a much younger, less-experienced workforce. We’ve had a huge influx of probably 40 or 50 staff at one of our prisons and they’re young and inexperienced and lots of the more experienced staff have left and are leaving… that’s a huge part to play because years back we used to work as a team, very much so, we were part of that team and we could rely on officers… but now it’s a very different way of working.”* (Professional participant)

**Tables 2-4: Patient safety incident report examples from the National Reporting and Learning System for applying the rating of avoidability of harm and the 2-tier assessment system**

**Table 2: cardiovascular disease**
Vignette highlighting the avoidability of harm where Tier 1 could be considered more avoidable than Tier 2. 65-year-old male. Past medical history of Myocardial Infarction, hypertension, diabetes, smoker, and body mass index (BMI) of 35. Prescribed Ramipril, Bisoprolol, glyceryl tri-nitrate (GTN) spray and diet-control for diabetes.

|  | **Cardiovascular disease** | **Tier 1 (Community)** | **Tier 2 (Prison context)** | **Contributory factors** |
| --- | --- | --- | --- | --- |
| 1 | **Totally unavoidable (Virtually no evidence of avoidability)** | Medical records appropriately coded with electronic reminders about patient characteristics and risk factors to notify healthcare professionals. The patient is on the Hypertension register, tagged for ‘Diabetes recall’ and the previous Myocardial Infarction is appropriately coded. Evidence in the notes that the patient has been recalled as per the evidence base for blood tests, medication, and blood pressure monitoring. | On arrival to the prison, the medical records from the community GP are received, reviewed, and transfer of salient information is completed to construct a full record suitable for care delivery in prison. Patient characteristics and known risk factors and coded within the system: they are on the hypertension register and the previous Myocardial infarction is coded. A task has been created by a nurse for a G.P to review medication and to ensure that blood pressure monitoring will take place. | No evidence of contributory factors in the notes. |
| 2 | **Unavoidable (Slight to modest evidence of avoidability)** | At routine appointments, despite documented risk factors (BMI/smoker),  neither smoking cessation or healthy lifestyle advice is discussed or documented within records. | Some evidence that lifestyle factors are recognised from a disease prevention perspective – nurse documents updated body mass index and summarises discussion about healthy lifestyle changes.  The patient is referred to the ‘Well man’ clinic and Nicotine Replacement Therapy is offered. | Opportunity for health promotion / disease prevention activities missed by the general practice (Tier 1). |
| 3 | **Possibly avoidable (Less than 50-50, but close call)** | Evidence in the notes that the patient has missed their annual blood pressure check. Reminder had been sent via a text message, but to an out-of-date mobile number on medical records – patient had not updated their details.  No follow up letter sent due to an administration error. | Blood pressure checked on arrival to prison and regularly within the ‘Well man’ clinic. A nurse arranged an annual blood test and bloods were taken within the prison and sent to the lab. | Poor protocols in the general practice (Tier 1)  with recall for annual blood pressure review. No procedure in place for ensuring patient contact details are checked periodically on contact with patients and kept up to date, nor a clear instruction on how patients can do this. Responsibility for maintaining contact details can be attributed to both patient and practice |
| 4 | **Probably avoidable (More than 50-50, but close call)** | Despite missing their blood pressure review, hypertension-related medication was not reviewed and re-authorised in error by a trainee. | After a review of blood tests and blood pressure, hypertension-related medication was updated by the medical team in line with NICE guidance on transfer into prison. | Poor protocols in the general practice (Tier 1) for medication review – the staff did not review medications appropriately and action following an important missed blood test was not taken. |
| 5 | **Probably avoidable (Strong evidence of avoidability)** | Patient attends the hospital for blood tests for diabetic annual review. Raised cholesterol and HBA1c noted. However, the diabetic nurse specialist was on annual leave, and there is no evidence in the notes that this was reviewed / actioned by the nurse.  Despite known risk factors and if calculated, a high Q risk, the patient is not offered/ prescribed a statin. The patient is subsequently missed off list for a diabetic review. | After blood tests were taken, a Q-risk was calculated by the healthcare team. Due to a high cholesterol, the patient is invited for discussion about statins and with their input, is prescribed a statin. | Poor protocols in the general practice (Tier 1) – review of blood results and related administration, appropriate calculation of cardiovascular risk. Poor recall for diabetic review. |
| 6 | **Totally avoidable (Virtually certain evidence of avoidability)** | Worsening hypertension, headaches, thirst, and frequent urination due to poorly controlled co-morbidities and lifestyle factors. Patient collapses and is admitted to hospital and has with a further myocardial infarction. | No progression of symptoms as both co-morbidities and lifestyle well controlled. Patient loses weight with controlled diet, exercise and appropriate primary and secondary prevention medications. | Protocols in the general practice (Tier 1) were insufficient to manage the patient’s cardiovascular risk factors. Evidence that co-morbid conditions not well managed as per evidence -based guidelines. |

**Table 3: Mental health.**

Vignette highlighting the avoidability of harm where Tier 2 could be considered more avoidable than Tier 1.

35-year-old male with a history of depression and previous admission under mental health section. Presented with worsening mood. Isolated, minimal support network and a history of both alcohol and substance misuse. Prescribed anti-depressants.

|  | **Mental health** | **Tier 1 (Community)** | **Tier 2 (Prison)** | **Contributory factors** |
| --- | --- | --- | --- | --- |
| 1 | **Totally unavoidable (Virtually no evidence of avoidability)** | Within community GP medical records, there is a documented past medical history of depression and suicidal ideation.  The patient has been asked to book a mental health review due to their repeat prescription for anti-depressants. A system is in place requiring the review prior to re-authorisation of further prescriptions. | On admission to prison, medical records are received from the patient’s previous general practice, they are reviewed, and transfer of information is completed to create a complete medical record. Previous history of depression and suicidal intent has been noted and documented in the notes on admission. | No evidence of contributory factors in the notes. |
| 2 | **Unavoidable (Slight to modest evidence of avoidability)** | Following a medication review and GP contact, there is evidence in the notes that an appropriate risk assessment has taken place. The doctor notes that the patient still admits to episodes of self-harm due to social circumstances and isolation. Frequent attendances to A+E are documented and evidenced by multiple discharge notifications, where the patient has been reviewed and discharged by the mental health team on each occasion. | The patient’s mood is noted as being low or deteriorating on multiple encounters with the healthcare team due to feeling isolated and following sentencing. A referral has been sent by the healthcare team to mental health team. The local protocol used by the prison healthcare team states the patient should be reviewed by the mental health team within one week. An appointment with the mental health team was issued for three weeks from the referral date due to their case load of patients. | Delay in appointment for mental health team (Tier 2) |
| 3 | **Possibly avoidable (Less than 50-50, but close call)** | After the patient’s recent suicide attempt, A+E arranged follow up with the local mental health liaison team for support. Despite multiple attempts, the patient did not answer follow up calls. Correct contact details on the system and the primary care team had been able to reach the patient for different reasons on subsequent dates. Letter sent to the patient’s address. | Following an episode of self-harm, an Assessment, Care in Custody and Teamwork (ACCT) document was appropriately initiated but the patient was not placed on the roster for regular observations. There is evidence a task was sent by the nursing team for GP to review in-possession medication, but medication was not removed automatically from the cell. | Insufficient follow up of the patient (Tier 1).  Insufficient observations, policies, and inadequate escalation with regards to in -possession medication (Tier 2) |
| 4 | **Probably avoidable (More than 50-50, but close call)** | The GP receives a letter from the mental health liaison team advising the patient has missed an appointment, and the GP documents that they have called the patient and spoken to him and members of his family. The GP arranges a consultation to discuss the patient’s recent suicide attempt, offers counselling and a review of medication.  The patient continues to self-harm. | The patient is suspected of taking an overdose of medication. It is documented that he has been acting erratically and has self-harmed within cell. The healthcare team were unable to take blood samples or perform an ECG that was clinically indicated due to a prison-wide lockdown and were not able to access prisoner. Concerns about the patient’s physical health were escalated to the Governor, but a decision was made not to admit at present due to lack of prison officers to act as an escort. The patient was monitored overnight and physiological observations were stable. | Prison priorities and organisational factors e.g., lockdown and secure environment, unable to access patient or admit to hospital.  Delay in assessment, management, and admission. Insufficient staff members to allow admission (Tier 2) |
| 5 | **Probably avoidable (Strong evidence of avoidability)** | Regular follow up appointments arranged with the GP, and crisis team interventions made. Weekly prescriptions of anti-depressant and community-based support including talking therapies offered. The patient continues to self-harm. | Due to full recovery from suspected overdose, admission to hospital is not arranged due to the upcoming mental health appointment at the prison and medications remain in – possession. | Insufficient follow up procedures following suspected overdose (Tier 2) |
| 6 | **Totally avoidable (Virtually certain evidence of avoidability)** | Despite support by the primary care team (general practice plus community mental health services), the patient took a further overdose with suicidal intent using prescribed medications and required admission to hospital. | The patient took a further overdose with suicidal intent. An admission to hospital was initially delayed due to insufficient staff numbers to act as a prison escorts, but they are admitted for observations. | Security vs healthcare priorities. Insufficient staff numbers to allow admission. Poor follow up of review of status of in-possession medications (Tier 2). |

**Table 4: Medication**

Vignette highlighting the avoidability of harm where there is equivalence between Tier 1 and Tier 2 considerations.

40-year-old with a history of epilepsy. Moved to the residential area and newly registered to the GP practice. The patient is prescribed multiple classes of anti-epileptic medication.

|  | **Mental health** | **Tier 1 (Community)** | **Tier 2 (Prison)** | Contributory factors |
| --- | --- | --- | --- | --- |
| 1 | **Totally unavoidable (Virtually no evidence of avoidability)** | The new patient registers at the GP practice. Medical records appropriately transferred from previous practice, and it is appropriately coded within the medical records that the patient has epilepsy. | New patient with background of epilepsy transferred into prison. Medical records from community-based general practice appropriately transferred, for full access. | No evidence of contributory factors in the notes. |
| 2 | **Unavoidable (Slight to modest evidence of avoidability)** | During registration at the GP surgery, the patient relayed correct information about their prescribed medication but incorrect doses were advised to the GP at a medication review.  Last correspondence from Neurology consultant was sent to the patient’s previous practice and this was not sent through when the patient’s notes were transferred. | Patient relayed correct medication names but incorrect doses were inputted by the GP at the initial assessment.  Last correspondence from Neurology consultant was sent to community-based GP practice. | Poor continuity of care, unable to access centralised system of letters. Patient mistake with doses. (Tier 1+2). |
| 3 | **Possibly avoidable (Less than 50-50, but close call)** | Incorrect doses of anti-epileptics prescribed without clarifying the correct doses with the specialist team. | Incorrect doses of anti-epileptics prescribed without clarifying the correct doses with the specialist team. | Poor continuity of care between primary and secondary care (Tier 1 and 2). |
| 4 | **Probably avoidable (More than 50-50, but close call)** | Medication prescribed at incorrect doses. Agreed to send prescription to local pharmacy that the practice has an agreement with to provide same day delivery. Pharmacy did not have the drug in stock, ordered it and delivered it to the patient two days later. | Medication was prescribed but the pharmacist noted the medication was not in stock. Medication was not available until the next day. | Delay with stock delivery (Tier 1).  Lack of stock and delay in ordering prescription (Tier 2). |
| 5 | **Probably avoidable (Strong evidence of avoidability)** | An incident report on the GP system indicates that the community pharmacy dispensed the incorrect medication due to confusion between branded and generic medications. | An incident report in the healthcare record states the prison pharmacy dispensed the incorrect medication due to confusion with branded and generic medications. | Medication related errors and mistake (Tier 1 + Tier2.) |
| 6 | **Totally avoidable (Virtually certain evidence of avoidability)** | The patient received incorrect medication dose for several days and had a prolonged fit requiring admission to hospital. | The patient received the incorrect medication dose for several days and had a prolonged fit and was admitted to hospital. | Medication error leading to harm. (Tier 1 +2) |
